# Identification and validation of a muscle failure index to predict prognosis and immunotherapy in lung adenocarcinoma through integrated analysis of bulk and single-cell RNA sequencing data

**DOI:** 10.3389/fimmu.2022.1057088

**Published:** 2023-01-17

**Authors:** Xuyu Gu, Lubing Cai, Zhiwen Luo, Luze Shi, Zhen Peng, Yaying Sun, Jiwu Chen

**Affiliations:** ^1^ School of Medicine, Southeast University, Nanjing, China; ^2^ Department of Sports Medicine, Shanghai General Hospital, Shanghai Jiao Tong University School of Medicine, Shanghai Jiao Tong University, Shanghai, China; ^3^ Department of Sports Medicine, Huashan Hospital, Fudan University, Shanghai, China

**Keywords:** lung adenocarcinoma, muscle failure index, ScRNA-seq, prognosis, immunotherapy

## Abstract

**Background:**

It was previously reported that the production of exerkines is positively associated with the beneficial effects of exercise in lung adenocarcinoma (LUAD) patients. This study proposes a novel scoring system based on muscle failure-related genes, to assist in clinical decision making.

**Methods:**

A comprehensive analysis of bulk and single cell RNA sequencing (scRNA-seq) of early, advanced and brain metastatic LUAD tissues and normal lung tissues was performed to identify muscle failure-related genes in LUAD and to determine the distribution of muscle failure-related genes in different cell populations. A novel scoring system, named MFI (Muscle failure index), was developed and validated. The differences in biological functions, immune infiltration, genomic alterations, and clinical significance of different subtypes were also investigated.

**Results:**

First, we conducted single cell analysis on the dataset GSE131907 and identified eight cell subpopulations. We found that four muscle failure-related genes (BDNF, FNDC5, IL15, MSTN) were significantly increased in tumor cells. In addition, IL15 was widely distributed in the immune cell population. And we have validated it in our own clinical cohort. Then we created the MFI model based on 10 muscle failure-related genes using the LASSO algorithm, and MFI remained an independent prognostic factor of OS in both the training and validation cohorts. Moreover, we generated MFI in the single-cell dataset, in which cells with high MFI received and sent more signals compared to those with low MFI. Biological function analysis of both subtypes revealed stronger anti-tumor immune activity in the low MFI group, while tumor cells with high MFI had stronger metabolic and proliferative activity. Finally, we systematically assessed the immune cell activity and immunotherapy responses in LUAD patients, finding that the low MFI group was more sensitive to immunotherapy.

**Conclusion:**

Overall, our study can improve the understanding of the role of muscle failure-related genes in tumorigenesis and we constructed a reliable MFI model for predicting prognosis and guiding future clinical decision making.

## Introduction

Lung cancer has the second highest incidence of all malignant tumors and is the leading cause of cancer-related death worldwide (1), with lung adenocarcinoma (LUAD) being the most common subtype (2). Although substantial progress has been made in tumorigenesis and LUAD therapy, the 5-year overall survival rate is still less than 20% (3), which apparently cannot meet satisfaction. In the past decade, the application of immunotherapy targeting immune checkpoints has significantly changed the treatment strategies of LUAD. Emerging biomarkers have now been used to predict immunotherapy responses, including PD-L1 expression and tumor mutation burden (TMB) (4, 5). However, these biomarkers fail to fully reflect the heterogeneous tumor microenvironment (4, 6), and therefore the immunotherapy could only benefit a limited amount of LUAD patients (7). Thus, understanding the crosstalk between tumor immune microenvironment and LUAD, as well as exploring novel biomarkers and predictive models, may be important means to improve therapeutic effectiveness.

Physical exercise has long been known to have associations with reduced mortality (8) and lower cancer incidence (9, 10), and recently has been increasingly prescribed as a non-pharmacological intervention to cancer patients. The mechanisms underlying exercise and cancer therapy are not yet entirely understood. Previous studies have revealed that exercise could trigger multiple systematic responses against tumor. Aerobic exercise could promote the mobilization and activation of tumor-infiltrating IL15Rα+ CD8 T cells, restricting pancreatic tumor growth and sensitizing pancreatic tumors to α-PD-1 therapy and chemotherapy (11). Meanwhile, additional physiological effects, such as improving tumor perfusion and vascularization, limiting intratumoral hypoxia, and increasing body temperature, may also modulate anti-tumor immunity and the tumor microenvironment (12).

In response to acute and chronic exercise, skeletal muscle is capable of releasing exerkines, which comprise a broad variety of hormones, metabolites (13), peptides (14), DNA, mRNA, microRNA, and other RNA species (15). These exercise-derived molecules participate in multisystemic regulatory feedback, like glucose homeostasis (16), muscle function (14) and anti-inflammatory responses (17), through endocrine, paracrine, and autocrine pathways. It was previously reported that the production of exerkines is positively associated with the beneficial effects of exercise in cancer patients. For example, muscle contraction during exercise leads to the release of exerkines like IL-15, IL-7, and IL-6. These exerkines regulate NK cells, which then contribute to a reduction in tumor growth (12). However, little is known about the relationship between exercise-mediated genes, exerkines, tumor microenvironment, and cancer prognosis.

Brain-derived neurotrophic factor (BDNF) plays a pivotal role in the development and plasticity of normal brain function *via* activating the TrkB (18). In addition, the Bdnf mRNA levels in skeletal muscle is a muscle damage biomarker, and physical exercise has been demonstrated as an efficient stimulus for BDNF synthesis in rodent skeletal muscle (19). The FNDC5 gene encodes a membrane protein, termed fibronectin type III domain-containing protein5, which could be proteolytically cleaved into a hormone called irisin (20). The muscles and adipose cells have been regarded the major source of irisin (21), and the post-exercise irisin secretion from muscles leads to the activation of browning and energy expenditure in white adipose tissues (20). IL-15 is a four-α-helix-bundle pro-inflammatory cytokine that plays an important role in stimulating both innate and adaptive immune responses (22). IL-15 mRNA level has been found increased in skeletal muscle groups dominated by type 2 fibers, especially after resistance exercise (23). Myostatin, as a member of the transforming growth factor β (TGF-β) superfamily, is a myokine that regulates skeletal muscle growth by inhibiting muscle hypertrophy (24). As genes that are strongly related with physical exercise and skeletal muscle, these four genes have been revealed to influence tumorigenesis and cancer prognosis directly or indirectly.

The development of single-cell RNA sequencing (scRNA-seq) technology and related data analysis methods has provided unprecedented opportunities to reveal the molecular characteristics of different immune cell populations in TME (25). Previous studies have reported that exploring gene expression profiles based on the molecular characterization of immune cells extracted from scRNA-seq data may be an effective way to predict prognosis and immunotherapeutic responses in cancer patients (25, 26). In this study, we first performed a comprehensive analysis of scRNA-seq of LUAD to understand the molecular features of four model genes (BDNF, FNDC5, IL15, MSTN) in LUAD. Next, an MFI (Muscle failure index) was constructed for predicting the prognosis of LUAD by bulk RNA-seq analysis. Additionally, the predictive ability of MFI was validated in databases from TCGA and GEO, and the relationship between MFI and immunotherapy responses in LUAD was investigated, which may provide insight for further individualized treatment.

## Methods

### Online database data extraction

The single-cell transcriptome dataset GSE131907 was collected from the GEO database (https://www.ncbi.nlm.nih.gov/geo/) and the data were processed using the 10x Genomics method, containing 58 sequencing cases from 44 patients. We selected 29 samples for further analysis, containing the normal lung tissues, early stage, advanced stage and brain metastatic cancer cases in the dataset. For detailed data processing and ethics, please refer to the original article (27).

Transcriptome RNA sequencing data, Mutect2 mutation data, HumanMethylation450 array, copy number variation (CNV) data and corresponding clinical information of TCGA-LUAD patients were downloaded from TCGA database (https://cancergenome.nih.gov/) using GDC API. A total of 492 LUAD samples were collected after excluding patients with loss of follow-up and missing clinical information. The raw FPKM sequencing data were normalized by TPM and used as a training cohort. Three mature LUAD cohorts were collected from GEO: dataset GSE30219 from the Affymetrix HG-U133 Plus 2.0 Array platform, dataset GSE72094 from the Rosetta/Merck Human RSTA Custom Affymetrix 2.0 platform, and dataset GSE72094 from the Illumina HumanWG-6 v3.0 expression beadchip. To prevent batch effects on the chips, we merged the three GEO datasets and log2 normalized the data, using the “sva” package’s combat function (28). **Figure S1** showed that the batch effect is well removed. A total of 615 LUAD metadata with complete clinical information were used as the validation cohort. We collected publicly available immunotherapy cohorts with complete clinical information and transcriptomic data, finally accepted a cohort of 298 advanced uroepithelial cancer patients receiving anti-PD-L1 immunotherapy (Imvigor210) (29), and a non-small cell lung cancer (NSCLC) cohort containing 27 patients treated with PD1 (GSE135222) (30).

### Single-cell RNA sequencing data analysis

The R package “Seurat” was used to process the scRNA-seq data (31). Briefly, cells with “min.cells< 3” and “min.features< 200” were excluded. After filtering cells with > 60% mitochondrial sequencing counts and nFeature_RNA > 7000, a total of 47822 cells were retained for further analysis. The dataset was then normalized using Seurat’s NormalizeData and ScaleData functions, and the Python package “scanpy” was used to visualize gene expression in different cell types. Cell types were identified according to the cell annotations provided in the original text. The R package “CellChat” was used to identify receptor-ligand interactions between cell clusters (32). 8 receptor-ligand interactions between cell clusters were identified at the molecular level, and the pathways of intercommunication were inferred. Receptor-ligand pairs with P-values<0.05 were screened to assess the molecular interaction network between cells.

### Construction and validation of MFI models

The TCGA cohort was used as the training model. Firstly, myokine/exerkine-related genes of interest were identified by correlation analysis (|cor|>0.4), details of which are provided in **Table S1**. One-way COX regression screened for independent prognostic factors among 884 myokine/exekine-related genes, those factors with P<0.01 were used for further analysis. The LASSO penalized Cox proportional risk model was used to identify the best prognostic model, and a 5-fold cross-validation was set to prevent overfitting. Considering the random sampling of cross-validation, 300 iterations were performed to identify the most stable prognostic model. The model with the highest frequency of occurrence was used as the final prognostic model and the MFI (Muscle failure index) was generated according to the formula:


$$MFI=\sum i\;Coefficient(mRNA_{i})\times Expression(mRNA_{i})$$

To assess the predictive ability of the risk scores in the training and validation sets, we used the “survcomp” R package to calculate the consistency C-index, with larger c-index indicating the prediction being more accurate (33). The high-MFI and low-MFI groups were divided based on the median MFI, and the prognostic value of the risk model was systematically assessed by Km survival curves, single- and multi-factor Cox regression, and time-dependent ROC curves.

### Functional enrichment and immune infiltration analysis

We performed ssGSEA analysis to assess the immune-related pathway activity of the samples based on previously published molecular markers *via* the R package “gsva”, details of which are provided in **Table S2**. We also performed GSEA analysis between the high and low MFI groups and screened for significant KEGG pathways with p<0.05. Functional enrichment of genes was achieved using the Metascape database (www.metascape.org/).

We estimated the infiltration abundance of 22 immune cell types in the tumor samples using the R package “CIBERSORT” (34). The immune activity and tumor purity of the samples were assessed by the Estimate algorithm. The Immunophenoscore (IPS) was calculated based on previous studies, and higher IPS indicated stronger immune activity of the samples (35).

Finally, we also collected the Homologous Recombition Defects (HRD) score, Intratumor Heterogeneity, indel neoantigens and SNV neoantigens of the samples, from the study of Thorsson et al (36).

### Dissecting the picture of genomic variation between two subgroups

To compare the differences in mutation burden between the high and low MFI groups, we processed the mutation data using the “maftools” R package (37). We first calculated the total number of mutations in the samples, then identified those genes with a minimum number of mutations >40, compared the differences in mutation frequency between the high and low MFI groups using chi-square tests, and visualized them using maftools. CNV data were processed by Gistic 2.0 on the Genepattern website, identifying significantly amplified and missing chromosomal segments, assessing CNV differences on chromosomal arms, and visualizing CNV results using the R package ggplot2.

### Evaluating risk models for immunotherapy response

To assess the immunotherapy responses of patients, we predicted the responses using the TIDE (http://tide.dfci.harvard.edu) web tool. In addition, the unsupervised subclass mapping algorithm (https://cloud.genepattern.org/gp/) was used to assess the responses to anti-PD1 and anti-CTLA-4 immunotherapy. Lastly, we validated the predictive efficacy of MFI in the immunotherapy cohort Imvigor21.

### Clinical samples collection

73 patients diagnosed with LUAD and treated at our hospital from September 2015 to December 2020 were selected from tumor tissue and adjacent normal tissue (at least 5 cm away from lung cancerous tissue), including 46 males and 27 females, with a median age of 49-79 years and a mean age of (64.9 ± 7.3) years. All patients were also followed up every three months for five years. Patient inclusion criteria: 1, all were diagnosed with LUAD by postoperative pathological examination; 2, all did not receive radiotherapy or chemotherapy before surgery; 3, clinical data were complete. Patient exclusion criteria: 1, combined with chronic systemic diseases; 2, combined with other malignant tumors. This study design was reviewed and approved by the Medical Ethics Committee of the Zhongda Hospital, Southeast University (No. 2021ZDSYLL090-Y01). In accordance with the principles of the Declaration of Helsinki. All patients provided and signed the informed consent.

### qPCR

Total RNA from tumor tissue and cells was used using RNAiso Plus (TAKARA, Otsu, Shiga, Japan) and Trizol LS Reagent (TAKARA, Otsu, Shiga, Japan), respectively. The reliability of the obtained RNA was then verified using formaldehyde denaturation electrophoresis assay to continue the subsequent experiments. Subsequently, reverse transcription polymerase chain reaction (RT-PCR) experiments were performed using the PrimeScript™ RT kit (TAKARA, Otsu, Shiga, Japan) strictly according to the instructions. The mRNA expression levels were quantified by standard real-time quantitative PCR methods, using SYBR Premix Ex Taq (TAKARA, Otsu, Shiga, Japan). GAPDH was used as a reference gene.

### Immunofluorescence

Sections were routinely dewaxed and rehydrated and incubated with PD-1 primary antibody (1:200, ABcam) and CTLA4 primary antibody (1:100, ABcam) at 4°C overnight. Cell climbing slides were washed with PBS and incubated with Alexa Fluora 594 or Alexa Fluora 488-labeled sheep anti-rabbit or secondary antibody (1:5000, ABcam) for 1h. The fluorescence intensity of PD-1 and CTLA4 was followed by DAPI nuclear fluorescence microscopy (Leica DM 3000).

### Bioinformatics and statistical analysis

All statistical analyses and graphing were performed using R software (version 4.04). The Wilcoxon test was used for the comparison between two groups, and differences in proportions were compared by chi-square test. The Kaplan-Meier plotter was used to generate survival curves and statistically significant differences were assessed using the log-rank test. Time-dependent ROC curves (tROC) were plotted using the R package “survivalROC”. Single- and multi-factor COX regressions were done using the R package “survival”. The R package “rms” was used to plot nomogram and calibration curves, and Decision curve analysis (DCA) was performed *via* the DCA package. Differences were considered significant at the values of two-tailed p< 0.05 unless especially specified.

## Results

### Identification of the expression profile of myokine/exerkine genes in LUAD

**Figure 1:**
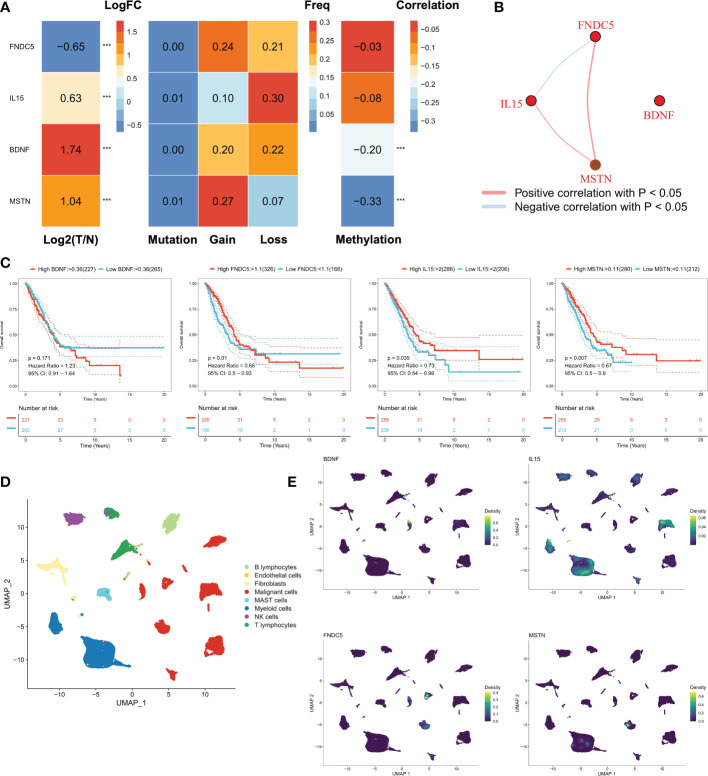
Genomic regulation of myosin genes in LUAD. **(A)** Transcriptomic profiles of the mRNAs of four secreted proteins, from left to right: analysis of the differences between tumor and normal samples, FNDC5 was down-regulated, while IL15, BDNF, and MSTN were up-regulated; mutations: all four genes had few mutations and mutations were not the main regulators of these genes; the frequency of chromosomal amplification and deletion at the corresponding loci could be seen to be substantial. In terms of genomic variation, these four proteins were mainly regulated by copy number variation, for example, the amplification number of MSTN was high, which may lead to its overexpression; methylation regulation, the methylation levels of BDNF and MSTN were negatively correlated with mRNA expression, and lower methylation levels may lead to the abnormal increase of BDNF and MSTN in tumor samples. **(B)** Correlation of the four genes, BDNF was not significantly correlated with the other three genes, while IL15 was negatively correlated with FNDC5 and positively correlated with MSTN; MSTN and FNDC5 were positively correlated. **(C)** The survival curves of four genes, FNDC5, IL15 and MSTN were all favorable factors for OS in LUAD patients, while BDNF had no significant effect on survival. **(D)** Cellular subpopulation of lung cancer single cell data, a total of 8 cellular subpopulations were identified according to the original annotation. **(E)** Distribution of four genes in different cell subpopulations, IL15 was mainly found in immune cells, while BDNF, MSTN and FNDC5 all mainly in malignant cells. ***P<0.001.

Our study focused on four myokine/exerkine genes (BDNF, FNDC5, IL15, MSTN). We first summarized the genomic regulatory profiles of myokine/exerkine genes in TCGA-LUAD patients (**Figure 1A**). These four genes showed significant expression differences between tumor and normal tissues, with FNDC5 downregulated in tumors, whereas BDNF, IL15 and MSTN upregulated in the tumor group. Further, rare mutations and extensive copy number variants (CNVs) of these genes were observed, suggesting that CNVs contributed to the regulation of these genes. For example, extensive copy number amplification of MSTN in tumors may lead to increased expression of MSTN in tumors. In contrast, in terms of IL15, mainly copy number deletion could be observed. Moreover, DNA methylation plays a regulatory role for BDNF and MSTN, lower methylation levels may cause an abnormal increase of BDNF and MSTN in tumor samples. We then analyzed the correlation of the four myokine/exerkine genes (**Figure 1B**), which showed that BDNF was not significantly correlated with the other three genes, while IL15 was negatively correlated with FNDC5 and positively correlated with MSTN; MSTN and FNDC5 were positively correlated. Subsequently, we found that IL15 and MSTN were favorable factors for OS in LUAD patients, while FNDC was an unfavorable factor for OS in LUAD patients. BDNF had no significant effect on survival (**Figure 1C**). We analyzed the dataset GSE131907 at single-cell resolution and identified a total of eight cell subpopulations according to the original annotation (**Figure 1D**). The four genes were apparently increased in tumor cells, and in addition, IL15 was widely distributed in the immune cell population (**Figure 1E**).

### The expression of four myokine/exerkine genes is associated with a poor prognosis in LUAD patients and with immune escape

**Figure 2:**
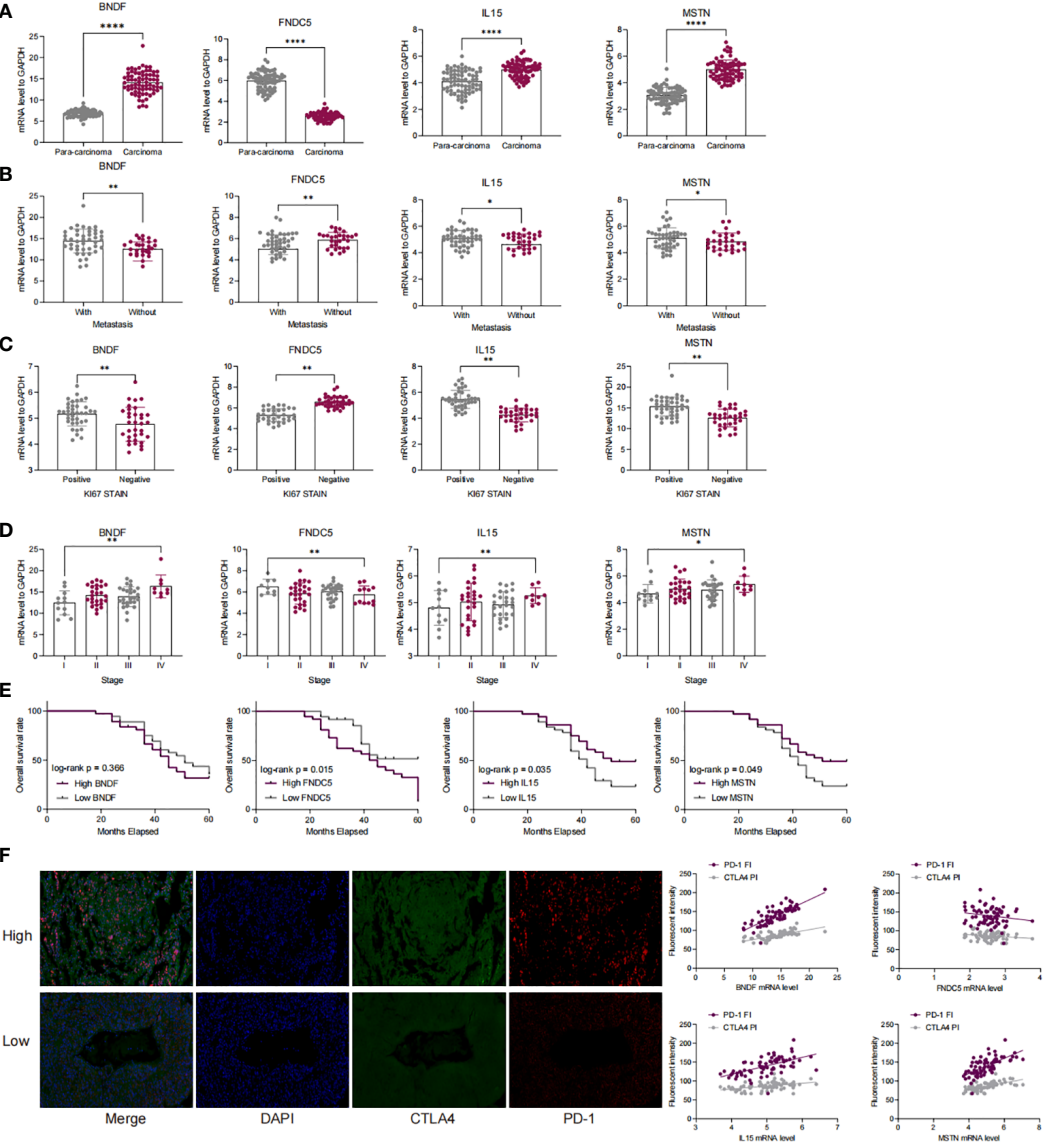
The expression of four myokine genes is associated with a poor prognosis in LUAD patients and with immune escape. **(A)** qRT-PCR detection of mRNA expression of BDNF, FNDC5, IL15, MSTN in cancer and paraneoplastic tissues; **(B–D)** Correlation of mRNA expression of BDNF, FNDC5, IL15, MSTN with lymph node metastasis, clinical stage, and ki67 in patients with LUAD; **(E)** Kaplan-Meier analysis of the correlation between the survival time in LUAD patients over 3 to 5 years and the expression levels of BDNF, FNDC5, IL15, and MSTN; **(F)** PD-1 and CTAL 4 was detected by double-label immunofluorescence and analyzed for the correlation of the fluorescence intensity of PD-1 as well as CTAL 4 with the expression of BDNF, FNDC 5, IL 15, and MSTN. *P<0.5, **P<0.01, ****P<0.0001.

We examined the mRNA expression of four myokine/exerkine genes, BDNF, FNDC5, IL15, and MSTN, in tumor and paraneoplastic tissues. The mRNA expression of BDNF, IL15, and MSTN were significantly higher in tumor tissues than in paraneoplastic tissues, while the expression of FNDC5 was significantly reduced in tumor tissues (**Figure 2A**). Further analyzing the correlation of these four genes for whether they had lymph node metastasis, whether they were positive for ki67, and the clinical stage of the patients according to the clinical baseline information of the patients, we found that high expression of the four genes was associated with the tumour progression of the patients with LUAD (**Figures 2B–D**). Moreover, we conducted a Kaplan-Meier analysis of the correlation between the expression of the four genes in patients’ survival over 3-5 years. The cut-off thresholds was the median level of BDNF, FNDC5, IL15, and MSTN mRNA level. And the results showed that patients with high expression of IL15 and MSTN and low expression of FNDC5, had a longer survival, while BDNF had no significant effect on survival (**Figure 2E**). In **Figure 2F**, we detected the staining intensity of PD1 and CTLA4 in tumor tissues by immunofluorescence, and analyzed the correlation between the mRNA levels of the four genes and the staining fluorescence intensity of PD1 and CTLA4. The results showed that high expression of BDNF, IL15, MSTN corresponded to high fluorescence intensity of PD1 and CTLA4, while the expression of FNDC5 was negatively correlated with the fluorescence intensity of PD1 and CTLA4.

### Construction and validation of MFI model

**Figure 3:**
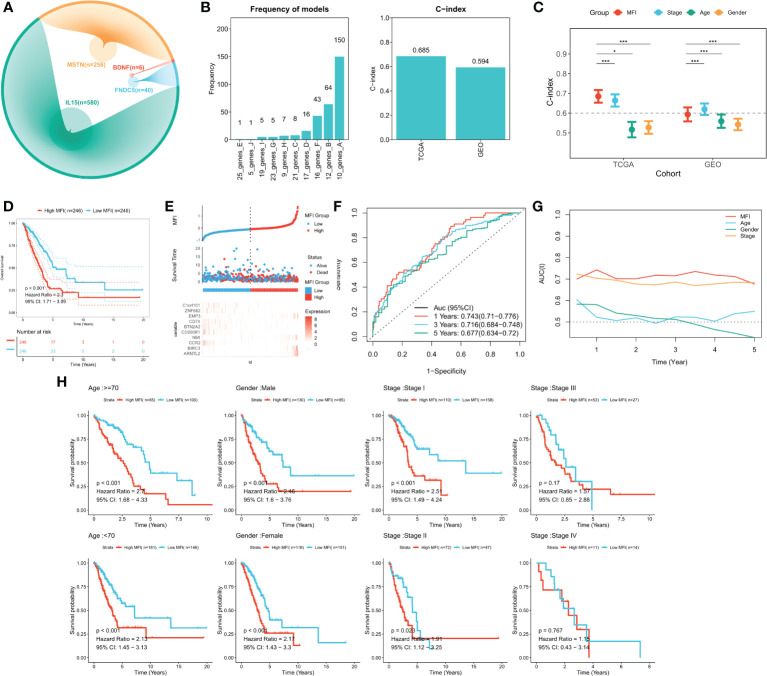
Building and validating the MFI model. **(A)** We identified genes with high correlation with our 4 model genes(r>0.4), finding 884 genes in total, including 580 of IL15, 40 of FNDC5, 6 of BDNF and 258 of MSTN. **(B)** Screening the best LASSO model, left: frequency of different gene combinations in the LASSO Cox regression model; right: C-INDEX of the best model in the TCGA and GEO cohorts. **(C)** Comparison of C-INDEX of different metrics, MFI had a leading advantage in TCGA. **(D)** Survival curves of high and low LIS subgroups, high MFI group showed significant worse survival. **(E)** Distribution of MFI in the TCGA cohort and transcriptional profiles of model genes. The scatter plot showed the survival status of patients with different LIS in the TCGA cohort. The low MFI group had fewer death cases and better survival. ARNTL2, BIRC3, NMI, EMP3 were favorable factors, whereas CCR2, CD200R1, BTN2A2, CD74, ZNF682, C1orf101 were adverse factors. **(F)** ROC plot of MFI in the TCGA cohort. The AUC values of the model at 1, 3, and 5 years were 0.743, 0.716, and 0.677, respectively. **(G)** tROC curve of MFI in the TCGA cohort. The results indicated that MFI is the best predictor. **(H)** Subgroup analysis suggested that MFI was applicable to patients in different ages and gender groups, while in terms of staging, it was mainly suitable for patients with early LUAD. *P<0.5, ***P<0.001.

We identified genes significantly associated with the four genes using pearson correlation analysis, and a total of 884 muscle failure-associated genes were identified according to a cor>0.4 threshold (**Figure 3A**). Detailed results are provided in **Table S2**. Next, 56 muscle failure-related genes were determined with significant prognostic efficacy using one-way cox regression analysis. We came up with the muscle failure-related genes related risk models using these 56 muscle failure-related genes and performed 300 iterations of LASSO regression. Among all ten combinations, we found that the model containing 10 genes was the most stable (150/300), and it had good accuracy in both the training and validation cohorts (TCGA:0.685; GEO:0.594) (**Figure 3B**). This LASSO model was constructed based on the optimal λ value of 0.0522, and the detailed model coefficients are provided in **Table S3**. We also constructed the MFI in the external validation cohort. Based on the C-index, we found that the MFI was a reliable predictor in both cohorts (**Figure 3C**). Patients at high and low risk were distinguished using the median MFI. Survival analysis suggested that patients in the high-risk group had lower survival than those in the low-risk group (**Figure 3D**; P<0.0001). **Figure 3E** reveals the distribution of MFI in the TCGA cohort and the transcriptional profiles of the model genes. The AUC values of the model at 1, 3, and 5 years were 0.743, 0.716, and 0.677, respectively (**Figure 3F**). tROC analysis indicated that MFI was the best predictor (**Figure 3G**). Subgroup analysis showed that MFI was suitable for patients of different ages and genders, while in terms of staging, it was mainly suitable for patients with early LUAD (**Figure 3H**). The predictive efficacy of the model was also evaluated in the validation set, with survival analysis suggesting a significantly worse survival of the high MFI group (**Figure S2A**, p<0.0001). **Figure S2B** shows the distribution of MFI and expression of model genes in the GEO cohort. ROC analysis exhibited adequate predictive efficacy of the model in the external validation set, specifically, 0.615 at 1 year, 0.624 at 3 years, and 0.583 at 5 years (**Figure S2C**). Subgroup analysis demonstrated that MFI was applicable in young and middle-aged patients (Age<70), both genders, and in early LUAD patients (**Figure S1D**).

### Assessing the predictive independence of MFI

**Figure 4:**
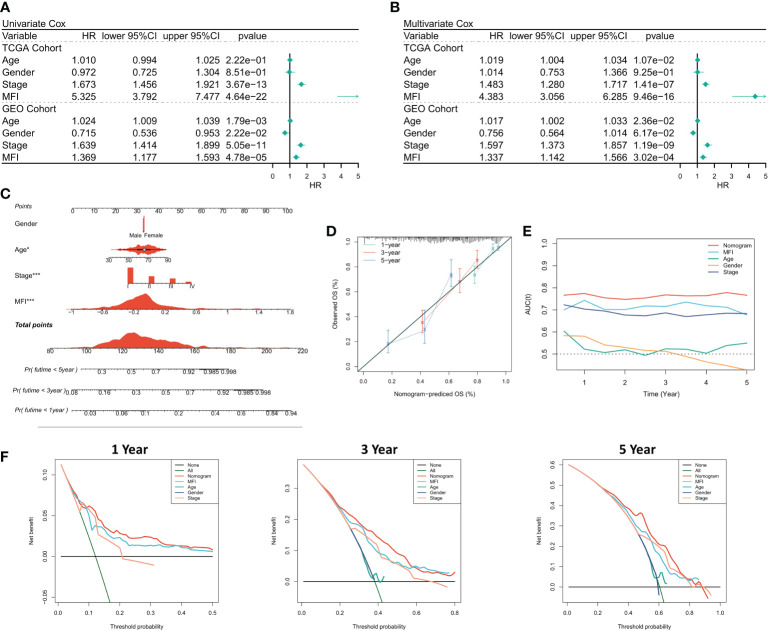
Assessing the predictive independence of MFI. **(A)** One-factor COX regression analysis of OS in TCGA and GEO cohorts. MFI was an independent prognostic indicator in both the training and validation sets; **(B)** Multi-factor COX regression analysis of OS in the TCGA and GEO cohorts. After correcting the other three common clinical variables, multifactorial cox still suggests MFI as a significant independent prognostic factor; **(C)** Quantification the risk of individual patients *via* Nomogram to predict the survival at 1, 3, and 5 years; **(D)** Calibration curves of Nomogram at 1, 3, and 5 years. The results revealed satisfied stability and accuracy of Nomogram model at 1, 3 and 5 years; **(E)** tROC curves of Nomogram. Nomogram model was a better predictor compared to clinical characteristics; **(F)** DCA curves of Nomogram at 1, 3 and 5 years.

We first analyzed the relationship among the risk score, clinical characteristics of the cancer patients, and their prognosis, using one-way Cox and multi-way Cox regression. In one-factor Cox regression, MFI was an independent prognostic indicator in both the training and validation sets (p<0.0001) (**Figure 4A**). Multi-factor Cox regression showed that, after correction for other clinical characteristics, MFI remained an independent prognostic factor for OS in both the training and validation cohorts (p<0.0001) (**Figure 4B**). Thus, we concluded that the risk score could be a reliable prognostic marker for OS in LUAD patients. We then constructed the Nomogram to better quantify risk assessment in LUAD patients (**Figure 4C**). Nomogram correction curves showed good stability and accuracy of the Nomogram model at 1, 3 and 5 years (**Figure 4D**). tROC analysis suggested that the Nomogram model was the best predictor compared to clinical characteristics (**Figure 4E**). We further performed DCA to evaluate the decision benefit of the Nomogram model, and it demonstrated that Nomogram was suitable for risk assessment of LUAD patients at 1, 3, and 5 years (**Figure 4F**).

### MFI at single-cell resolution

**Figure 5:**
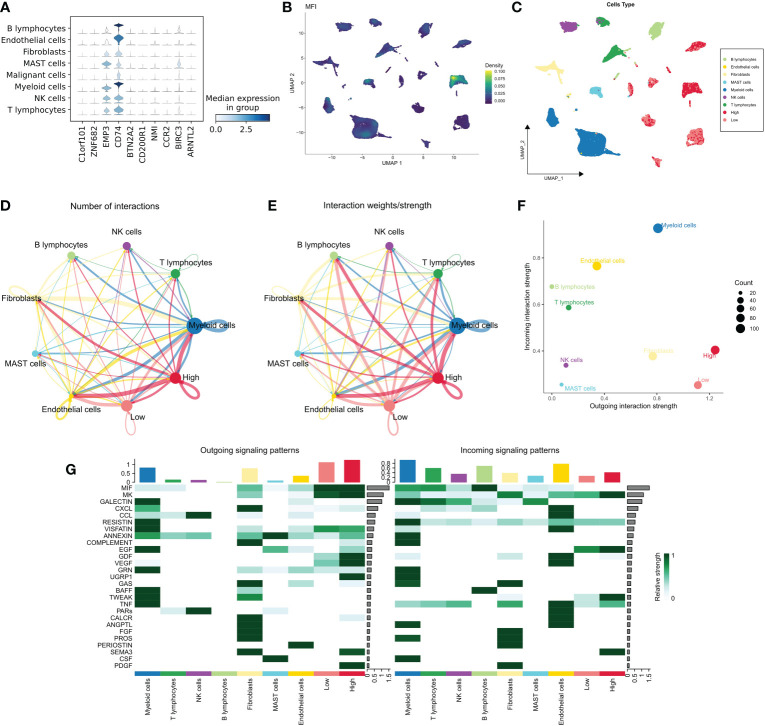
MFI at single-cell resolution. **(A)** Distribution of the model genes in different cells, showing that EMP3, CD74 and BIRC3 were widely distributed in immune-related cell populations and less expressed in malignant cells. The remaining 7 model genes were less expressed in all cell types; **(B, C)** Distribution of MFI in different cells. MFI was mainly distributed in a specific group of malignant cells and most of the immune cells **(B)**; we divided the malignant cells into high and low MFI groups based on MFI **(C)**; **(D, E)** The cellular communication network of different cells. High MFI tumor cells had more receptor-ligand communications; **(F)** The number of communications in different cells, High MFI tumor cells receive and send more signals, indicating that these cells are more active; **(G)** Evaluation of specific communication pathways. Our results showed that compared to low MFI cells, high MFI cells were more active in GDF, VEGF, SEMA3 and UGRP1 pathways.

We examined the distribution of MFI model genes in different cell populations and found that EMP3, CD74 and BIRC3 were widely distributed in immune-related cell populations and less expressed in malignant cells. The remaining 7 model genes were relatively silent in all cell types (**Figure 5A**). Further, we generated MFIs in the single-cell dataset, which revealed that MFIs were mainly allocated in a specific group of malignant cells as well as in most immune cells (**Figure 5B**). We classified malignant cells into high and low MFI groups based on MFI (**Figure 5C**). To understand the functional differences of these groups, we performed cellular communication analysis. The communication networks between all cells are shown in **Figure 5D** and **Figure 5E**, with high MFI cells receiving and sending more signals (**Figure 5F**). Finally, we inferred the specific pathways of intercellular communication and found that high MFI cells showed stronger activity in the GDF, VEGF, SEMA3 and UGRP1 pathways compared to the low MFI group (**Figure 5G**). These results suggest that high MFI cells could be characterized as more active in cancer proliferation, metabolism, and angiogenesis.

### Differences in the biological functions of the two subtypes

**Figure 6:**
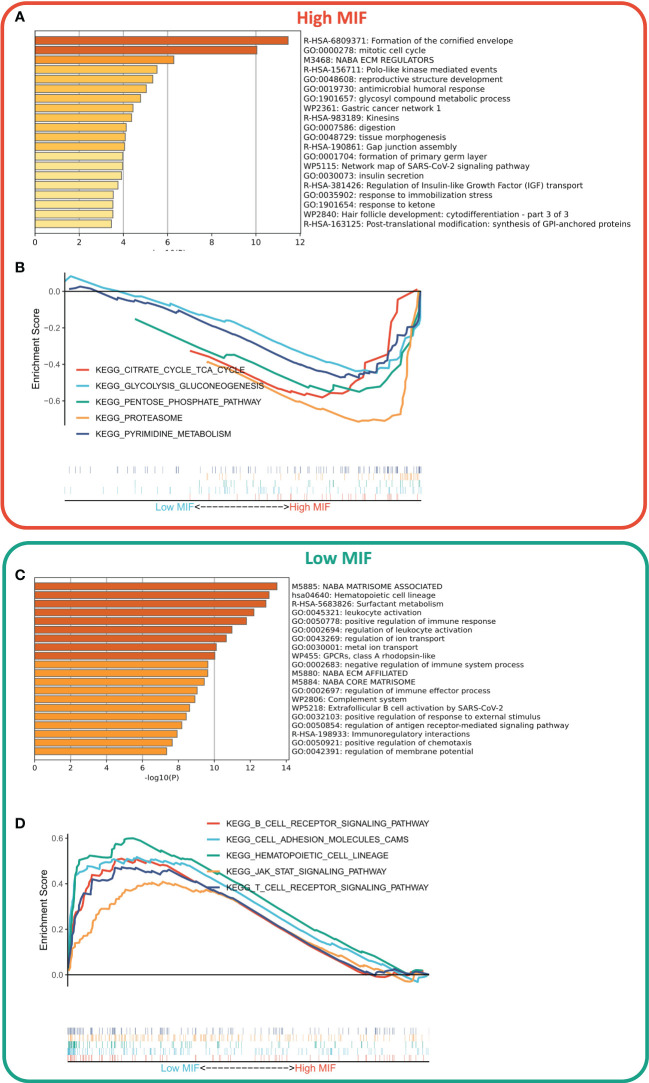
Differences in biological functions of the two subtypes. **(A, C)** GO analysis of high- and low-expressed genes. Functional enrichment analysis showed that the upregulated genes in the high MFI group mainly regulated cell cycle and Gap-linked pathways **(A)**, while the upregulated genes in the low MFI group mainly regulated hematopoietic cell lineage, inflammatory responses, cell adhesion and lymphocyte migration **(C)**; **(B, D)** KEGG analysis of high and low-expressed genes. The enriched pathways in the high MFI group were TCA cycle pathway, pentose phosphate pathway, proteasome, and glycolytic pathway **(B)**. In contrast, the pathways that were enriched in the low MFI group are mainly B-cell receptors, T-cell receptors and Jak-stat signaling pathways, as well as hematopoietic cell line-related pathways **(D)**.

We first identified DEGs between the two subtypes *via* limma package, and a total of 806 DEGs were identified according to the threshold of FDR<0.05, FC>2, of which 607 DEGs were upregulated in the high MFI group and 199 DEGs were upregulated in the low MFI group. Functional enrichment analysis showed that the upregulated genes in the high MFI group mainly regulated cell cycle and Gap-linked pathways (**Figure 6A**), while those upregulated in the low MFI group mainly regulated hematopoietic cell lineage, inflammatory response, cell adhesion and lymphocyte migration (**Figure 6C**). GSEA analysis indicated that the enriched pathways in the high MFI group were TCA cycle pathway, pentose phosphate pathway, proteasome, and glycolytic pathway (**Figure 6B**). On the contrary, the pathways that were enriched in the low MFI group were mainly B-cell receptors, T-cell receptors and Jak-stat signaling pathways, as well as hematopoietic cell line-related pathways (**Figure 6D**). In conclusion, these results confirm that the low MFI group has stronger anti-tumor immune activity, whereas the high MFI tumor cells tend to have stronger metabolic and proliferative activity, which may contribute to the differences in prognosis between the two groups.

### Dissecting the immune infiltration of MFI

**Figure 7:**
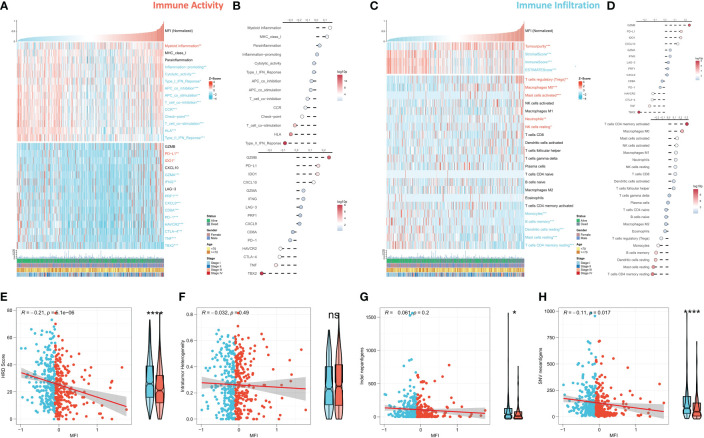
Differences in immune infiltration between the two subtypes. **(A, B)** Assessing the relationship between MF, MFI, immune pathway activity, typical immune checkpoints and clinical features in terms of immune activity. Tumor myeloid immunity, PD-L1 and IDO1 were significantly increased in the high MFI group and positively correlated with MFI. On the contrary, the activity of all immune pathways except MHC Class 1 and paracancerous immunity was significantly increased in the low MFI group, and almost all immune checkpoints were negatively correlated with MFI and increased in the low MFI group. **(C, D)** The relationship between MFI, MFI, Estimate score, immune cell abundance and clinical features was evaluated from the perspective of immune infiltration. The results revealed increased tumor purity in the high MFI group, while stromal cells and immune cells increase in the low MFI group. In addition, Tregs, M0 macrophages, neutrophils and mast cells increased in the high MFI group, while monocytes, DC cells, memory B cells and CD4 memory T cells increased in the low MFI group. **(E–H)** Differences between the two groups in four indicators related to tumor-specific antigens: HRD score, Indel neoantigens, Intratumor Heterogeneity and SNV neoantigens. The results showed a significant negative correlation between MFI and HRD score, Indel neoantigens and SNV neoantigens, and the three indexes were significantly increased in the low MFI group; however, there was no significant correlation between MFI and Intratumor Heterogeneity, which indicated more chromosomal instability in the low MFI group and more tumor neoantigens with stronger immune system activity, suggesting that low MFI group tend to benefit more from immunotherapy. ns p>0.05; *p< 0.05; ****p< 0.0001.

We further evaluated the correlation between MFI and immune landscape in detail. Initially, we assessed MFI in terms of immune activity, and the heat map demonstrates the relationship between MFI, immune pathway activity, typical immune checkpoints, and clinical features (**Figure 7A**). The corresponding correlation analysis is shown on the right side of the heat map (**Figure 7B**). The results demonstrated that myeloid immunity, PD-L1 and IDO1, were significantly increased in the high MFI group and positively correlated with MFI. Conversely, all immune pathway activities except MHC Class 1 and paracancerous immunity were significantly increased in the low MFI group, and almost all immune checkpoints were negatively correlated with MFI and enhanced in the low MFI group. We next evaluated MFI from the perspective of immune infiltration, and the heat map shows the relationship between MFI, Estimate score, immune cell abundance and clinical features (**Figure 7C**). The corresponding correlation analysis is shown on the right side of the heat map (**Figure 7D**). The results indicated increased tumor purity in the high MFI group, whereas stromal cells and immune cells increased in the low MFI group. In addition, we observed an increase of Tregs, M0 macrophages, neutrophils and mast cells in the high MFI group, while monocytes, DC cells, memory B cells and CD4 memory T cells were increased in the low MFI group. Finally, we analyzed four indicators related to tumor-specific antigens: HRD score, Indel neoantigens, Intratumor Heterogeneity and SNV neoantigens (**Figures 7E–H**). The results revealed that MFI has a negative correlation between HRD score, Indel neoantigens and SNV neoantigens, and these three indicators were increased in the low MFI group. Intratumor Heterogeneity, however, showed no significant correlation with MFI, indicating the low MFI group is characterized with more severe chromosome instability, more tumor neoantigens, and stronger immune activity, suggesting this group may be more likely to benefit from immunotherapy.

### Correlation between MFI and genomic variation

**Figure 8:**
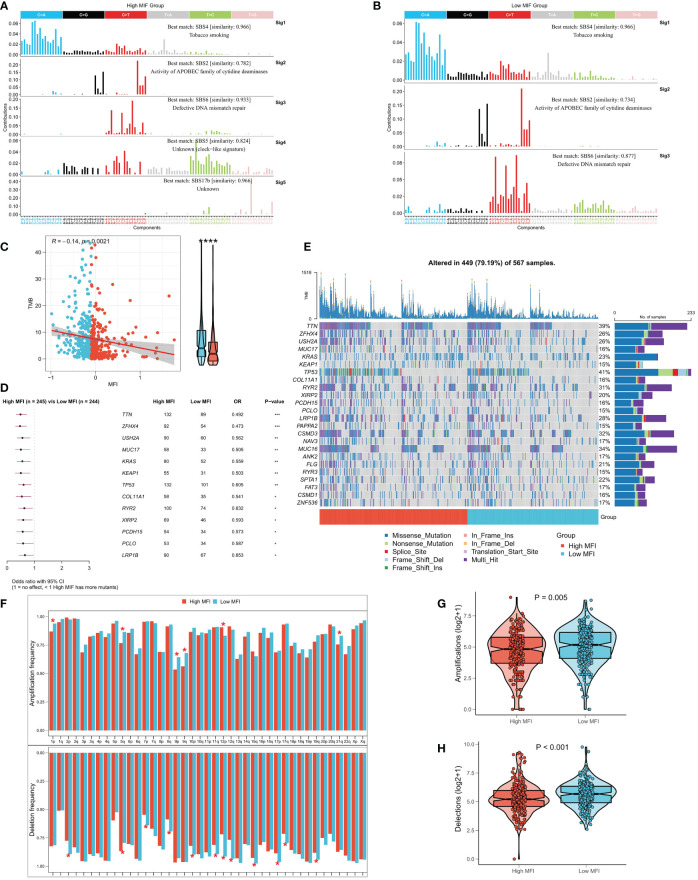
Correlation between MFI and genomic variation. **(A, B)** Mutation marker events in the high and low MFI groups, showing that smoking, APOBEC and DNA mismatch repair-related mutation events (SBS4, SBS2 and SBS6) were frequent in both high and low MFI groups. **(C)** Differences in TMB between the two subtypes, showing that MFI was significantly negatively correlated with TMB, and the latter was significantly increased in the low MFI group. **(D)** Forestplot showed the driver mutated genes with significantly higher mutation frequency in the low MFI group. **(E)** Oncoplot showed the mutation landscape of TOP24 mutation driver genes among subtypes. **(F)** Histogram revealed the CNV events on chromosome arms among subtypes. **(G)** Differences in overall amplification events between subtypes. **(H)** Differences in overall deletion events between subtypes. *P<0.5, **P<0.01, ***P<0.001, ****P<0.0001.

Several recent studies have observed that TMB is related with immunotherapy responses, which may due to the increased mutation-derived antigens generated by somatic cell mutations. When the immune system recognizes those antigens containing mutant peptides, anti-tumor immunity would then be activated. Considering the critical role of TMB, we explored the correlation between TMB and MFI. We summarized the mutational signature events in the high and low MFI groups. The results showed that smoking, APOBEC and DNA mismatch repair-related mutational events (SBS4, SBS2 and SBS6; **Figures 8A, B**) occurred frequently in both high and low MFI groups. Notably, two unknown mutational events, SBS5 and SBS17b, also presented in high MFI group, suggesting that SBS5 and SBS17b may be cancer markers of that associated with muscle failure. Afterwards, we found a negative correlation between MFI and TMB, and TMB was significantly elevated in the low MFI group (**Figure 8C**). Forestplot shows the driver mutated genes with higher mutation frequency in the low MFI group (**Figure 8D**), and **Figure 8E** shows the mutational landscape of high frequency mutated genes in LUAD patients in detail. CNV has been known to cause chromosomal variation in another way, thus we further evaluated the relationship between MFI and CNV. We found an increased frequency of amplifications and deletions at the chromosome arm level in the low MFI group (**Figure 8F**). Box plots showed an increase in amplification and deletion events in the low MFI group (**Figures 8G, H**).

### Low MFI group is more sensitive to immunotherapy

**Figure 9:**
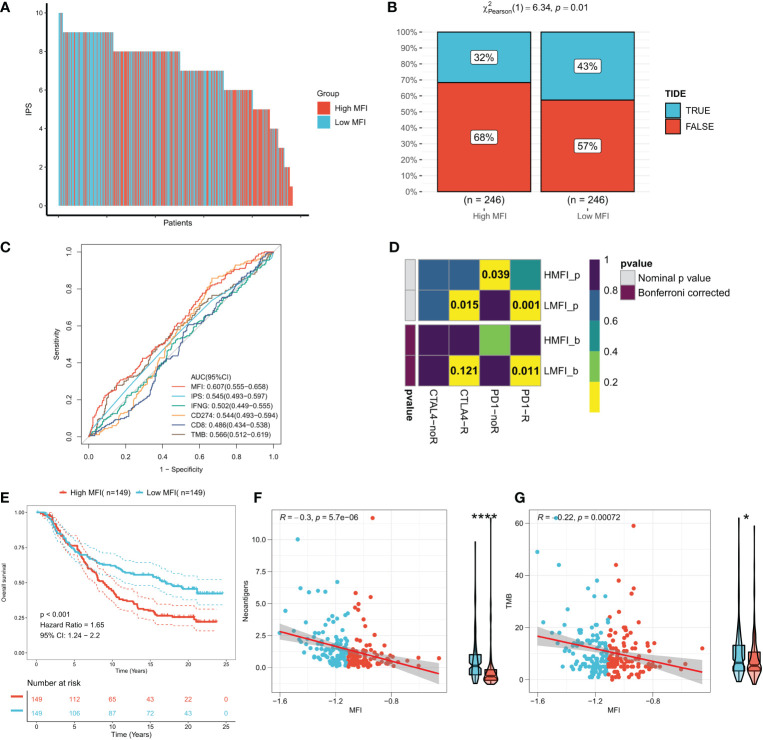
Low MFI group is more sensitive to immunotherapy. **(A)** Differences in IPS between the two subtypes. Low MFI group was characterized with apparently higher IPS; **(B)** IDE algorithm predicted the immunotherapy response rates in high and low MFI groups, showing that patients with low MFI had higher response rates (p=0.01); **(C)** ROC curves showed the predictive accuracy of MFI and different immunomarkers. Compared to other assessments, MFI showed a non-negligble advantage (AUC=0.607); **(D)** Subclassmapping assessed immunotherapy responses from another different perspectives. **(E)** Survival curves for high and low MFI subgroups in the Imvigor210 cohort. **(F)** Correlation of MFI with Neoantigens in the Imvigor210 cohort. **(G)** Correlation between MFI and TMB in the Imvigor210 cohort. *P<0.5, ****P<0.0001.

IPS can systematically assess effector immune cell activity and immunotherapy responses in cancer patients, and we found significantly higher IPS in low MFI group in both discovery TCGA and GEO cohorts (**Figure 9A**, **Figure S3A**). Meanwhile, the TIDE algorithm predicted that patients with low MFI may have higher response rates to immunotherapy (P=0.01, **Figure 9B**). ROC analysis showed that MFI exhibited a leading advantage over commonly used evaluation indicators (AUC=0.607, **Figure 9C**), and the same result was observed in the validation cohort (**Figures S3B, C**). Furthermore, subclass mapping results in both the TCGA and GEO cohorts suggested that patients in the low MFI group were more sensitive to anti-PD1 therapy (TCGA: FDR=0.011, GEO: FDR=0.027) (**Figure 9D**; **Figure S3D**). Patients with low MFI were also found to benefit from anti-CTLA-4 treatment in the TCGA cohort (P=0.015, **Figure 9D**). We constructed MFI in a well-established immunotherapy cohort and showed significantly worse survival in the high-risk group (P<0.001, **Figure 9E**). We then evaluated the relationship between TMB, neoantigens and MFI in the immunotherapy cohort and observed that MFI was negatively correlated with TMB and neoantigen number, with TMB and neoantigen number significantly higher in the low-risk group (**Figures 9F, G**), which may lead to a better outcome of PD-L1 immunotherapy. In conclusion, our results determined that low MFI group is more sensitive to immunotherapy.

## Discussion

As immunotherapy advances, a growing number of predictive biomarkers of immunotherapy responses are being identified (38). The impact of TME on cancer immunotherapy efficacy has been intensively studied, and TME-related biomarkers are now attracting more attention (39). However, reliable biomarkers and models focusing on tumorigenic TME for immunotherapeutic response and prognosis in LUAD still remain rare (40). The development and progression of scRNA-seq technology provide us a way to comprehensively understand the molecular signature of tumor-infiltrating immune cells in TME (41). It was previously reported that the production of myokine/exerkines is positively associated with the beneficial effects of exercise in cancer patients (42, 43). However, little is known about the relationship between exercise-mediated genes, exerkines, tumor microenvironment, and cancer prognosis. Here, we used single-cell RNA sequencing data to identify the expression profile of myokine/exerkine genes in LUAD and constructed the MFI model in Bulk sequencing data based on interacting genes. The significance of MFI in terms of clinical significance, biological function, TME and genomic alterations were also systematically assessed.

We first focused our perspective on the dynamic changes caused by muscle injury in LUAD and developed the MFI model, which could accurately predict the OS in patients with LUAD. Several researchers have previously proposed models for the risk of apoptosis, injury and stress-related LUAD (44–47).Our model showed better accuracy than previously reported methods, which was confirmed by comparing the AUC values. Moreover, our model could also be used to classify “cold” and “hot” tumors, which results in differences in genomic alternation status and sensitivity toward immunotherapy. To summarize, our results reveal for the first time the possible impact of muscle damage on the tumor microenvironment and show exciting clinical applications of LUAD.

We first identified the expression profiles of four genes (BDNF, FNDC5, IL15, MSTN) in LUAD. FNDC5 was down-regulated in tumors, while BDNF, IL15 and MSTN were up-regulated. Then, we examined the mRNA expression levels of BDNF, FNDC5, IL15, and MSTN, in tumor tissues and paracancerous tissues. The expression levels of BDNF, IL15, and MSTN were significantly higher in tumor tissues than in paraneoplastic tissues, while the expression level of FNDC5 was significantly reduced in tumor tissues. Next, we analyzed the dataset GSE131907 at single cell resolution and identified a total of 8 cell subpopulations according to the original annotation, finding that IL15 was mainly distributed in immune, whereas BDNF, MSTN and FNDC5 mainly distributed in malignant cells. We next generated the MFI model based on muscle failure-related genes by the LASSO algorithm, which showed excellent predictive efficacy in both the training dataset and the two external validation datasets, with a significantly lower survival rate for high-risk patients. Further, we analyzed of the correlation between the expression of the four genes in patients’ survival over 3-5 years, and the results showed that patients with high expression of IL15 and MSTN and low expression of FNDC5 had a longer survival. In addition, we detected the staining intensity of PD1 and CTLA4 in tumor tissues by immunofluorescence, and analyzed the correlation between the mRNA levels of the four genes and the staining fluorescence intensity of PD1 and CTLA4. The results showed that high expression of BDNF, IL15, MSTN corresponded to high fluorescence intensity of PD1 and CTLA4, while the expression level of FNDC5 was negatively correlated with the expression level of PD1 and CTLA4. The results suggest that the expression of four myokine genes is associated with poor prognosis and immune escape in patients with LUAD.

Further, we analyzed the distribution of MFI model genes in different cell populations, and the results showed that MFI mainly distributed in a specific group of malignant cells as well as in most immune cells. To understand the functional differences between different MFI subtypes of malignant cells, we performed cellular communication analysis, which showed that high MFI cells receive and send more signals compared to low MFI cells. We inferred specific pathways of intercellular communication, and the results suggest that high MFI cells are a group of cells that are more active in cancer proliferation, metabolism, and angiogenesis.

Functional enrichment analysis showed that the MFI group had stronger anti-tumor immune activity, while tumor cells with high MFI had stronger metabolic and proliferative activity, which may contribute to the differences in prognosis between the two groups. Moreover, immune infiltration analysis also suggested the presence of much severe chromosomal instability and more tumor neoantigens with stronger immune system activity in the low MFI group, suggesting that immunotherapy could bring more benefits to this group.

Next, to elaborate the molecular features of the two subtypes, we compared the genomic alterations. We first summarized the mutational signature events in the high and low MFI groups and found that smoking, APOBEC and DNA mismatch repair-related mutational events (SBS4, SBS2 and SBS6) showed high frequency in both high and low MFI groups. Interestingly, two unknown mutational events, SBS5 and SBS17b, also presented in high MFI group, suggesting that SBS5 and SBS17b may be muscle failure-associated mutational markers of cancer. MFI was significantly negatively correlated with TMB, which significantly elevated in the low MFI group. We further evaluated the correlation between MFI and CNV and observed an enhanced frequency of amplifications and deletions in the low MFI group at the chromosome arm level. Some studies have reported a negative correlation between CNV and the benefit rate of immunotherapy, which is contrary to our results and may be caused by more TMB producing more neoantigens (48, 49). These results suggest that the low-MFI group may have more tumor-specific neoantigens and showed promoted sensitivity to immunotherapy.

Finally, considering the heterogeneity of subtypes in immunotherapy, we evaluated the predicting efficacy of MFI. The results suggested that MFI exhibited high accuracy in the immunotherapy cohort, and it also showed better accuracy compared to commonly used biomarkers (MDSC, MSI score, IFNG, CD8 and CD274). We found that patients with low MFI may have more neoantigens, which may lead to greater sensitivity to immunotherapy. In conclusion, our results define that MFI is not only a robust prognostic marker, but also a promising predictive marker for immunotherapy.

There are several limitations of our study. First, this signature was constructed based on a public dataset. Its predictive ability needs to be further validated in a large prospective clinical study. Second, this study only assessed the predictive ability of MFI indirectly, without examining those patients receiving immunotherapy in real. Finally, this study did not include any *in vitro* or *in vivo* evidence to further explore the potential molecular mechanisms of MFI in predicting prognosis and immunotherapeutic response.

In summary, we proposed and validated a new prognostic model consisting of four muscle failure-related genes in LUAD, *via* a comprehensive analysis of single cells and bulk RNA sequencing. The model could serve as a valid prognostic biomarker and potentially predict the immunotherapy responses in LUAD patients. Our study provides new insights into the role of myokine/exerkine genes in the prognosis and immunotherapeutic responses of patients with LUAD.

## Data availability statement

The original contributions presented in the study are included in the article/**Supplementary Material**. Further inquiries can be directed to the corresponding authors.

## Ethics statement

This study design was reviewed and approved by the Medical Ethics Committee of the Zhongda Hospital, Southeast University (No. 2021ZDSYLL090-Y01). The patients/participants provided their written informed consent to participate in this study.

## Author contributions

XG, LC, and ZL carried out data organization and analysis. LS and ZP wrote results. YS organized the manuscript. JC proposed the idea and was responsible for the whole project. All authors contributed to the article and approved the submitted version.

## Supplementary material

Supplementary Figure 1 Removal of the batch effect. **(A)** The Umap plot shows a significant batch effect before removal. **(B)** The Umap plot shows no significant batch effect after removal.

Supplementary Figure 2 External validation of MFI. **(A)** Survival curves of the high and low MFI subgroups in the GEO cohort. **(B)** Scatter plot showing the survival status of patients with different MFI in the GEO cohort. **(C)** ROC analysis of MFI in the GEO cohort. **(D)** Subgroup analysis of MFI in patients with different clinical characteristics in the GEO cohort.

Supplementary Figure 3 Validation of subtype immune infiltration. **(A)** Differences in IPS between the two subtypes in the GEO cohort. **(B)** Using TIDE algorithm in the GEO cohort to predict the response rate to immunotherapy for high and low MFI groups. **(C)** ROC curves in the GEO cohort showing the predictive accuracy of MFI and different immune markers. **(D)** Prediction of immunotherapy response rates for high and low MFI groups in the GEO cohort *via* Subclassmapping algorithm.
