# Supplementary figures and images for: Identification and validation of a muscle failure index to predict prognosis and immunotherapy in lung adenocarcinoma through integrated analysis of bulk and single-cell RNA sequencing data
Source: Front Immunol. 2023 Jan 17;13:1057088. doi: 10.3389/fimmu.2022.1057088 (PMC9888242; doi:10.3389/fimmu.2022.1057088)

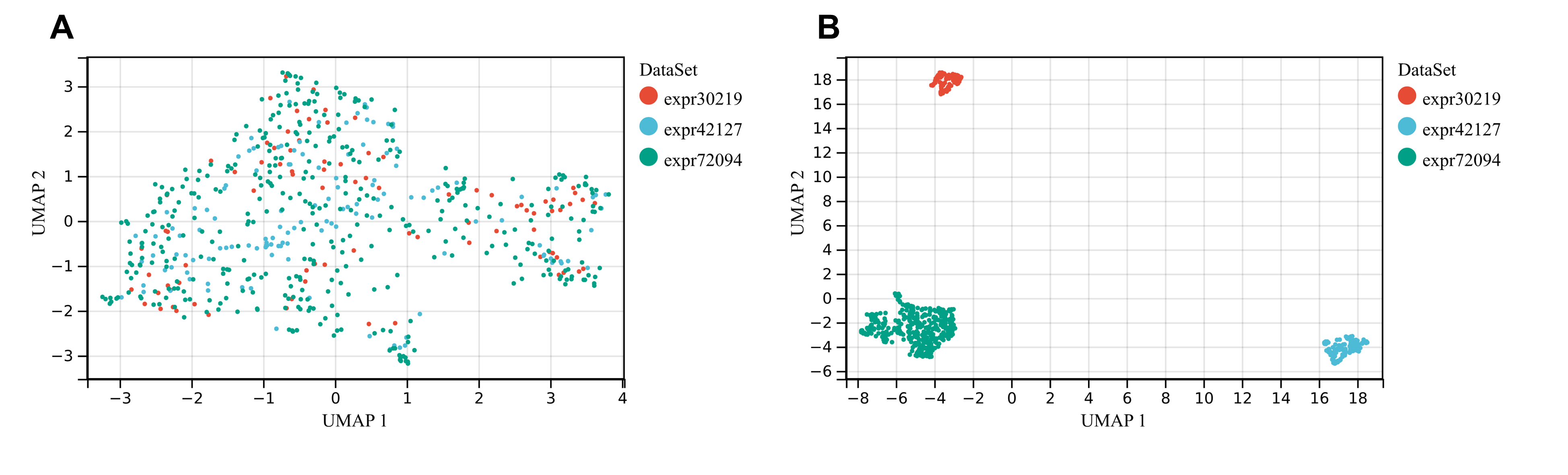

Supplement: Supplementary Figure 1 — Removal of the batch effect. (A) The Umap plot shows a significant batch effect before removal. (B) The Umap plot shows no significant batch effect after removal. [file Image_1.tif]

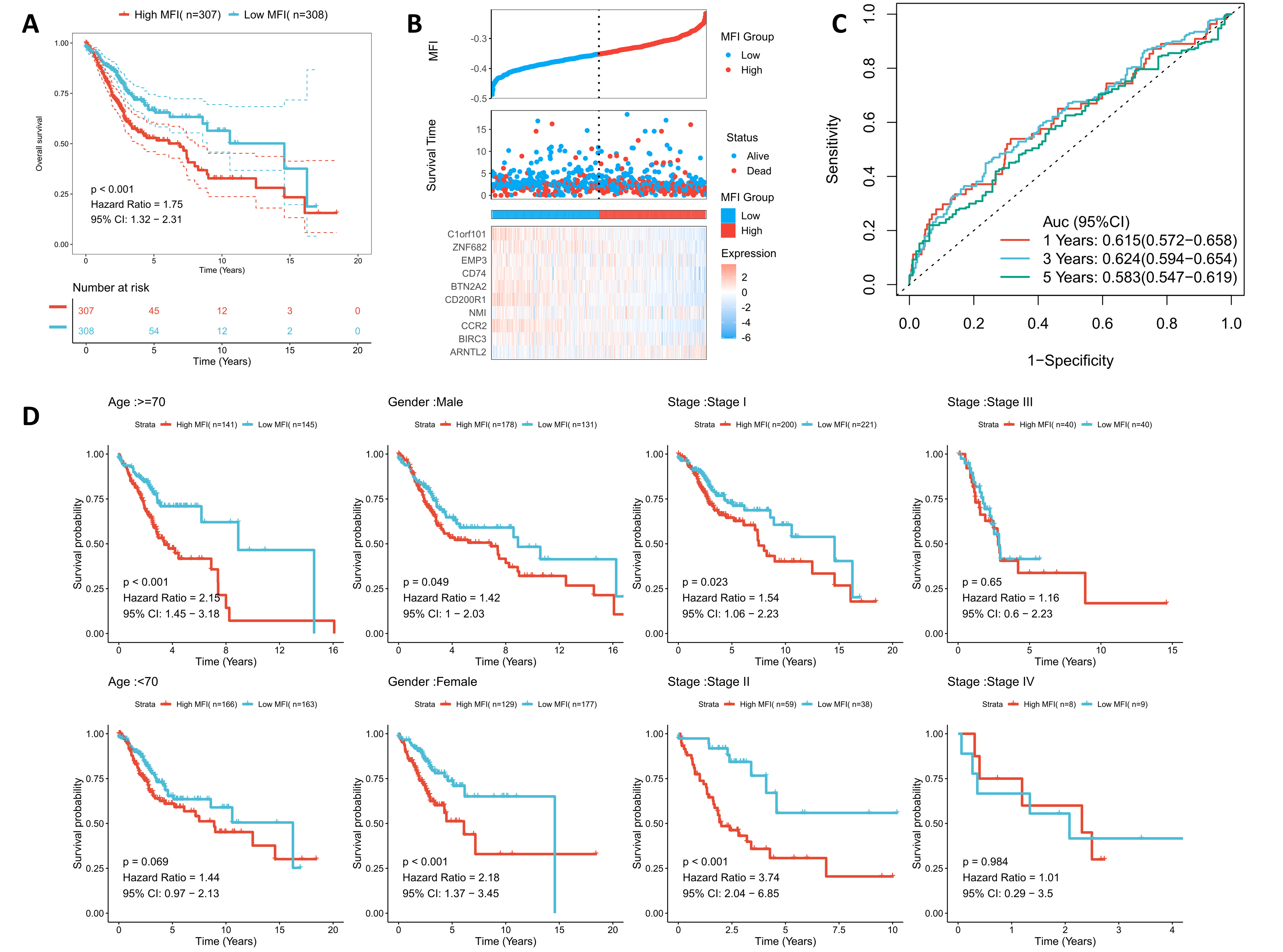

Supplement: Supplementary Figure 2 — External validation of MFI. (A) Survival curves of the high and low MFI subgroups in the GEO cohort. (B) Scatter plot showing the survival status of patients with different MFI in the GEO cohort. (C) ROC analysis of MFI in the GEO cohort. (D) Subgroup analysis of MFI in patients with different clinical characteristics in the GEO cohort. [file Image_2.tif]

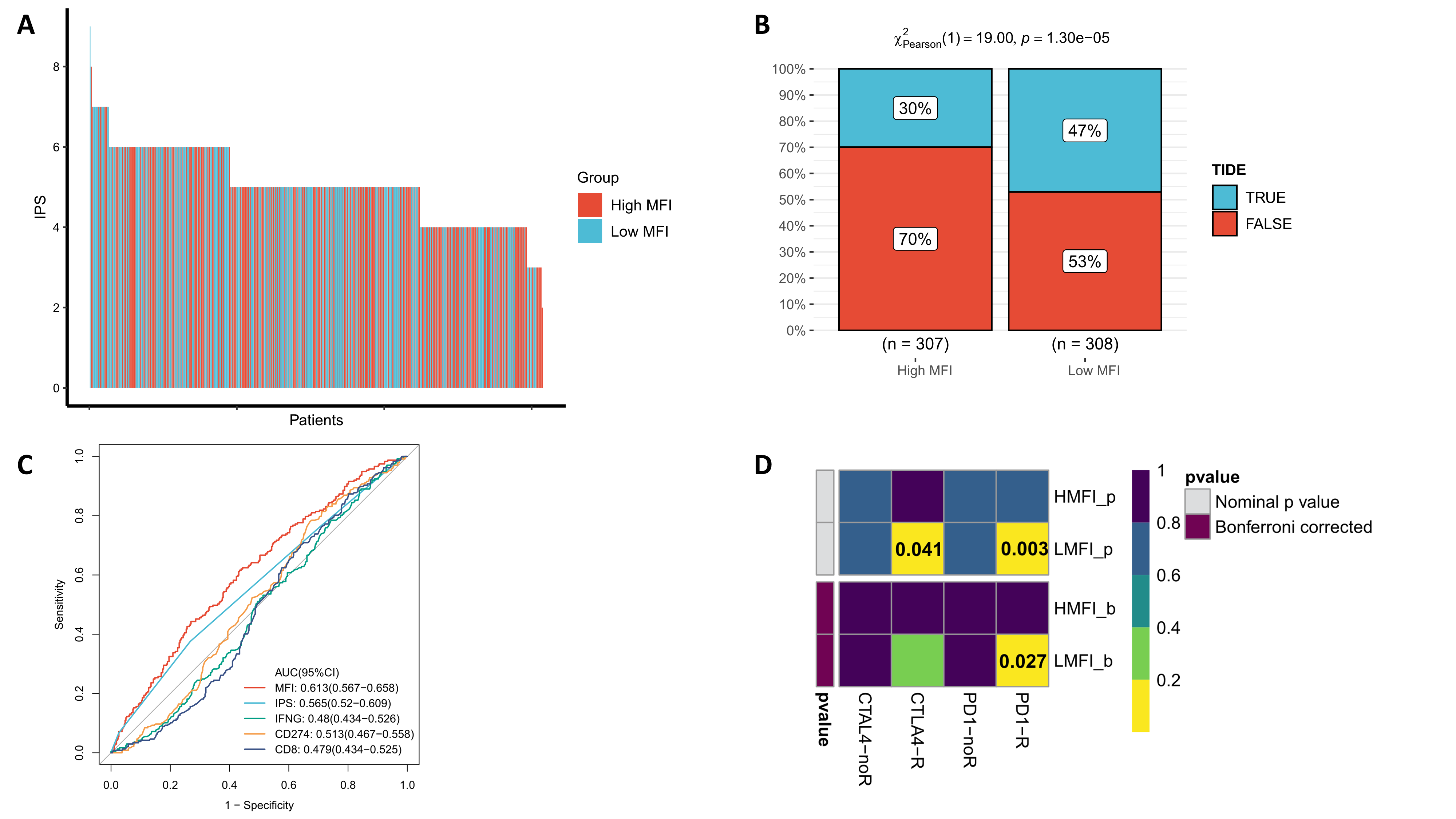

Supplement: Supplementary Figure 3 — Validation of subtype immune infiltration. (A) Differences in IPS between the two subtypes in the GEO cohort. (B) Using TIDE algorithm in the GEO cohort to predict the response rate to immunotherapy for high and low MFI groups. (C) ROC curves in the GEO cohort showing the predictive accuracy of MFI and different immune markers. (D) Prediction of immunotherapy response rates for high and low MFI groups in the GEO cohort via Subclassmapping algorithm. [file Image_3.tif]
